# Spatial early warning signals for impending regime shifts: A practical framework for application in real‐world landscapes

**DOI:** 10.1111/gcb.14591

**Published:** 2019-04-01

**Authors:** Jelmer J. Nijp, Arnaud J.A.M. Temme, George A.K. van Voorn, Lammert Kooistra, Geerten M. Hengeveld, Merel B. Soons, Adriaan J. Teuling, Jakob Wallinga

**Affiliations:** ^1^ Soil Geography and Landscape group, Department of Environmental Sciences Wageningen University & Research Wageningen the Netherlands; ^2^ KWR Watercycle Research Institute, Ecohydrology Group Nieuwegein the Netherlands; ^3^ Geography Department Kansas State University Manhattan Kansas; ^4^ Biometris Wageningen University & Research Wageningen the Netherlands; ^5^ Laboratory of Geo‐information Science and Remote Sensing, Department of Environmental Sciences Wageningen University & Research Wageningen the Netherlands; ^6^ Ecology and Biodiversity group, Institute of Environmental Biology, Biology Department Utrecht University Utrecht the Netherlands; ^7^ Hydrology and Quantitative Water Management Group, Department of Environmental Sciences Wageningen University & Research Wageningen the Netherlands

**Keywords:** alternative stable states, critical slowing down, early warning signals, ecosystem resilience, environmental change, landscapes, regime shifts, remote sensing, spatial patterns, tipping points

## Abstract

Prediction of ecosystem response to global environmental change is a pressing scientific challenge of major societal relevance. Many ecosystems display nonlinear responses to environmental change, and may even undergo practically irreversible ‘regime shifts’ that initiate ecosystem collapse. Recently, early warning signals based on spatiotemporal metrics have been proposed for the identification of impending regime shifts. The rapidly increasing availability of remotely sensed data provides excellent opportunities to apply such model‐based spatial early warning signals in the real world, to assess ecosystem resilience and identify impending regime shifts induced by global change. Such information would allow land‐managers and policy makers to interfere and avoid catastrophic shifts, but also to induce regime shifts that move ecosystems to a desired state. Here, we show that the application of spatial early warning signals in real‐world landscapes presents unique and unexpected challenges, and may result in misleading conclusions when employed without careful consideration of the spatial data and processes at hand. We identify key practical and theoretical issues and provide guidelines for applying spatial early warning signals in heterogeneous, real‐world landscapes based on literature review and examples from real‐world data. Major identified issues include (1) spatial heterogeneity in real‐world landscapes may enhance reversibility of regime shifts and boost landscape‐level resilience to environmental change (2) ecosystem states are often difficult to define, while these definitions have great impact on spatial early warning signals and (3) spatial environmental variability and socio‐economic factors may affect spatial patterns, spatial early warning signals and associated regime shift predictions. We propose a novel framework, shifting from an ecosystem perspective towards a landscape approach. The framework can be used to identify conditions under which resilience assessment with spatial remotely sensed data may be successful, to support well‐informed application of spatial early warning signals, and to improve predictions of ecosystem responses to global environmental change.

## Graphical abstract

Ongoing global change steadily increases pressure on ecosystems, to the point where a small additional change may cause a sudden ecosystem state shift and drastically modify ecosystem functioning. Predicting such generally undesired ‘regime shifts’ is crucial for timely management intervention. A promising prediction method is the translation of spatial vegetation patterns observed with, for example, satellite imagery into ecosystem resilience indicators. While these spatial indicators perform well in theory, we show how application to real‐world landscapes presents unexpected challenges and may even lead to misleading conclusions. Our study identifies the conditions for successful application of spatial indicators to real‐world landscapes.

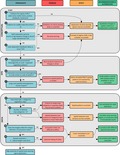

## 1 INTRODUCTION

Identification and prediction of ecosystem responses to global environmental change is currently a key scientific challenge (Sato & Lindenmayer, 2018; World Resources Institute, 2005). Given that ecosystems and societies are intimately linked, it is crucial to maintain ecosystem functioning within the safe operating space, the global and local stressors under which favourable ecosystem functioning and services can be secured (Scheffer et al., 2015). Ecosystems may respond gradually to increasing environmental pressures, such as climate change, or they may undergo a sudden ‘regime shift’ (also referred to as catastrophic shift or critical transition; Scheffer, Carpenter, Foley, Folke, & Walker, 2001). In the latter case, the services ecosystems provided for society remain relatively stable under increasing environmental pressure, but may suddenly shift towards a functionally different state after a threshold pressure is exceeded. Regime shifts are difficult to reverse due to positive feedbacks maintaining each of the alternative states, resulting in hysteresis (Box 1; Scheffer et al., 2001).

Box 1 **How spatial patterns may inform us on impending regime shifts**

1 To illustrate how spatial patterns may help identify looming regime shifts, we use the well‐established Noy‐Meir model (1973, 1975) that describes the nonlinear and hysteretic response of vegetation biomass to changing grazing pressure (*c*). We employed a spatially explicit extension of the Noy‐Meir model following Guttal and Jayaprakash (2009) that incorporates diffusive seed dispersal and random variation in space and time of the mean grazing rate (Supporting Information S1). The use of the Noy‐Meir model is intended for grassland ecosystems, but its concepts are applicable to other systems under pressure with two trophic levels.

**Figure B1:**
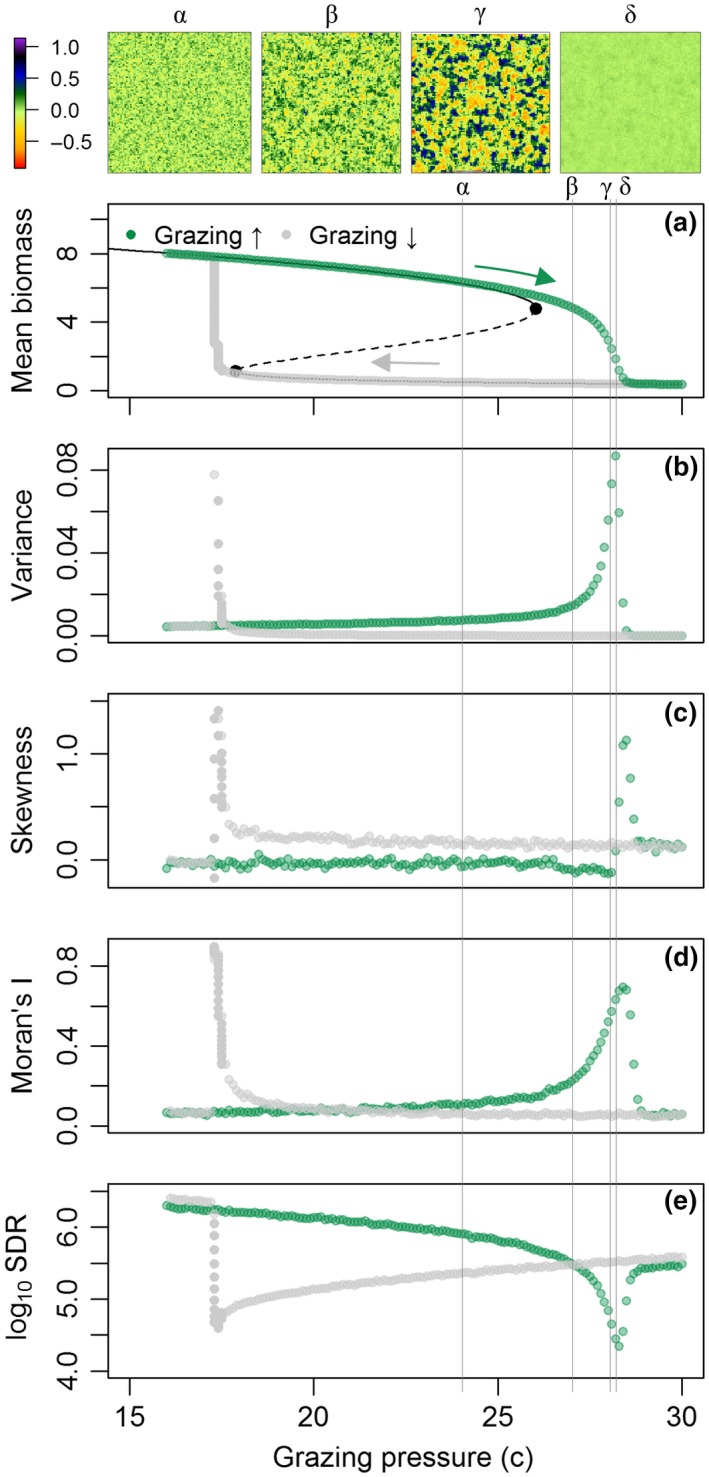
(a) Two alternative regimes of biomass as function of increasing and decreasing grazing pressure. The black curved lines represent stable (solid) and unstable (dashed) equilibria, the black dots indicate tipping points. Greek symbols and vertical lines indicate specific (increasing) grazing levels for which spatial snapshots are shown (α‐δ). The snapshots show deviations from mean biomass in the snapshot as variation in biomass was much larger between snapshots than within. (b–e) Metrics that are suggested to provide spatial early warning signals for regime shifts (following Kéfi et al., 2014). SDR is the spectral density ratio, defined as the ratio of spectral ‘power’ in the lowest (0%–20%) to highest (80%–100%) portion of spectral frequencies [Colour figure can be viewed at http://wileyonlinelibrary.com]

Clearly two alternative regimes of mean field biomass appear in response to grazing. Dependent on initial conditions, either of the two states is possible at grazing rates 18 < *c* < 26 (Figure B1a).

**Figure B2:**
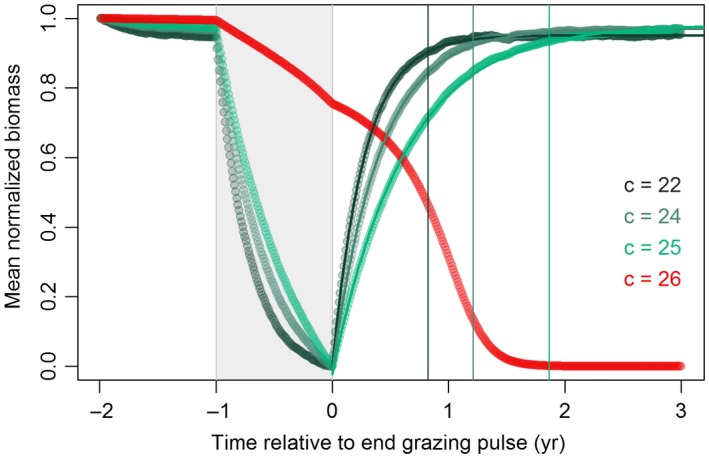
Biomass recovery after pulse events of increased grazing pressure slows down with lower resilience**.** A pulse of increased grazing pressure (Δ*c* = +1.0, duration = 1 year) was employed at different grazing levels to simulate recovery from perturbations. Biomass (*B*) was range‐normalized ${B^{\text{'}}}_{t=i}=\left(B_{t=i}-B_{min}\right)/\left(B_{min}-B_{max}\right)$ to ease comparison of recovery from perturbations at different grazing pressures. Recovery time was estimated from a saturated exponential function: $\left(t\right)=p_{1}\left(1-exp\left(-t/p_{2}\right)\right)+p_{3}$, where *B′* is the range‐normalized biomass, *t* is time, and *p*_1_, *p*_2_ and *p*_3_ are fitting parameters. Recovery time (vertical coloured lines) was defined as 95% of the asymptotic value, that is, 3·*p*_2_ conform Webster and Oliver (2007). All fits were significant (*p* « 0.01) and in good agreement with model‐generated data (Willmott's index of agreement > 0.99; Willmott et al., 1985) [Colour figure can be viewed at http://wileyonlinelibrary.com]

While a fully deterministic model provides useful information on stress–response relationships, there will always be small perturbations around the mean driver value in the real world. Under a continuous regime of stochastic events in driving variables, ecosystems constantly respond to perturbations. At low resilience, ecosystems take longer to recover from perturbations (Figure B2) as the strength of positive feedbacks diminishes (van Nes & Scheffer, 2007). This phenomenon is referred to as critical slowing down (Strogatz, 1994). Critical slowing down (CSD) is expressed by increased temporal autocorrelation and rising variance, thus representing EWS of regime shifts (Dakos et al., 2012).

In ecosystems with spatial interactions and large spatial connectivity, the increased recovery time associated with reduced resilience not only becomes expressed in temporal characteristics, but also in spatial patterns (Kéfi et al., 2014).

At low resilience, sites that have—by chance—large biomass have stronger positive feedbacks and therefore shorter recovery time as compared to low‐biomass sites. With accumulating stress (i.e. perturbations), high‐biomass sites thus remain high in biomass, whereas the biomass at low‐biomass sites reduces due to the low recovery rates. This phenomenon increases spatial variability (variance), often in combination with peaking spatial skewness (Scheffer et al., 2009) (Figure B1b–c). In addition, the importance of spatial interactions relative to local positive feedbacks increases at low resilience (Dakos et al., 2010). In the Noy‐Meir model example, seed dispersal will continue to occur as long as a spatial biomass gradient exists, whereas the (net) growth rate of existing vegetation reduces with increasing grazing pressure. As a result, vegetation cover will become more spatially structured with increased grazing pressure, as expressed by increasing Moran's I correlation and decreasing spectral density ratio (Figure B1d–e). These SEWS quantify changes in configuration that occur with reduced ecosystem resilience, and hence may be used to monitor resilience and detect impending regime shifts.

Regime shifts may have disastrous effects on numerous socio‐ecological systems around the world. In semi‐arid regions, reduced rainfall may turn woodland vegetation irreversibly into bare soil (Rietkerk et al., 2002), thereby affecting societies that depend on wood cover for food, fuel and construction material (International Energy Agency, 2018). In northern peatlands, changes in rainfall may induce shifts towards reduced carbon storage regimes (Hilbert, Roulet, & Moore, 2000), hence accelerating global climate change. Conversely, regime shifts may also provide opportunities for conservation of socio‐ecological systems by inducing shifts towards desired ecosystem states with relatively limited interference (Pueyo et al., 2008; Ripple & Beschta, 2012; Scheffer, 1998).

Given the potentially detrimental socio‐ecological consequences of regime shifts, the development of indicators to predict upcoming regime shifts has evolved as a major theme. Such indicators are often referred to as Early Warning Signals (EWS) (Scheffer et al., 2009). Model analyses suggest that impending regime shifts can be detected with EWS based on time series of ecological data, without system‐specific mechanistic knowledge on potential drivers (Dakos et al., 2012; Scheffer et al., 2009). A major drawback of such EWS, however, is that long‐term, uninterrupted and high‐resolution data records are needed (Scheffer et al., 2009). Lack of such data often hampers the application of temporal EWS to detect socio‐ecological systems at risk.

Recent theoretical advances suggest that metrics describing the spatial organization of ecosystem patterns, derived from spatially gridded data obtained from remotely sensed imagery or extensive field inventories, may also function as EWS (Box 1). Such Spatial Early Warning Signals (SEWS) including patch size distributions (Kéfi et al., 2007), spatial variance and skewness (Guttal & Jayaprakash, 2009), spatial autocorrelation (Dakos, van Nes, Donangelo, Fort, & Scheffer, 2010), wavelength analyses (Carpenter & Brock, 2010), recovery length (Dai, Korolev, & Gore, 2013), cross‐scale connectivity (Zurlini, Jones, Riitters, Li, & Petrosillo, 2014), spatial heteroscedasticity (Seekell & Dakos, 2015) and Fisher information (Sundstrom et al., 2017). Compared to temporal indicators, SEWS have the advantage that they can be applied on spatial data with irregular and infrequent temporal resolution (Génin, Majumder, Sankaran, Danet et al., 2018). The increasing availability and resolution of remotely sensed gridded data (Gómez, White, & Wulder, 2016) therefore provides a unique opportunity to monitor ecosystem resilience and detect impending regime shifts induced by global change all around the globe.

SEWS are increasingly applied to infer resilience of ecosystems (e.g. Berdugo, Kéfi, Soliveres, & Maestre, 2017; Butitta, Carpenter, Loken, Pace, & Stanley, 2017; Cline et al., 2014; Eby, Agrawal, Majumder, Dobson, & Guttal, 2017; Kéfi et al., 2007; van Belzen et al., 2017; Weerman et al., 2012). But while prospects and limitations of SEWS have been demonstrated conceptually using simulation models (e.g. Dakos, Carpenter, van Nes, & Scheffer, 2015; Dakos, Kéfi, Rietkerk, van Nes, & Scheffer, 2011; Guttal & Jayaprakash, 2009), empirical validation in real‐world examples remains scarce (Kéfi et al., 2014). The application of SEWS in real‐world landscapes comes with abundant conceptual and practical challenges. In contrast to the idealized systems used in models to assess the functioning of SEWS, landscapes are in reality complex adaptive systems that vary in space due to geomorphological processes and where, especially in the Anthropocene, socio‐ecological interactions can no longer be neglected (Sterk, van de Leemput, & Peeters, 2017). The complex adaptive systems where ecological, societal and geomorphic factors interact are hereafter, for simplicity, referred to as real‐world landscapes. Arguably, the landscape perspective on SEWS is crucial to enhance their applicability in real‐world landscapes.

**Figure 1:**
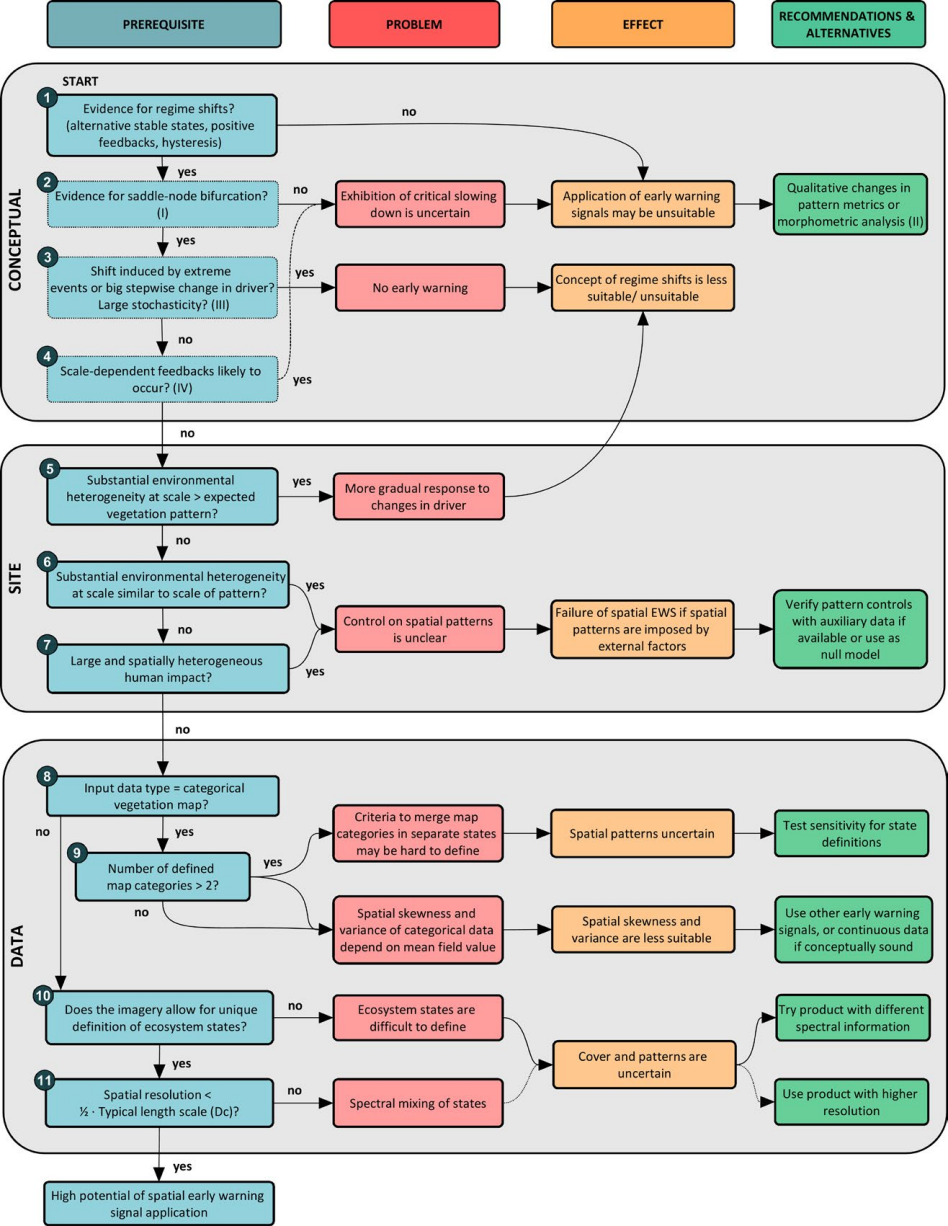
Prerequisites for application of spatial early warning signals to infer impending regime shifts in real‐world applications. The framework indicates main problems, their cause and effect on spatial early warning signals and regime shift prediction, and options to solve these problems. The numbered prerequisites refer to section numbers. Prerequisites with thin‐lined boxes are based on published conceptual reviews on early warning performance. Roman numerals refer to: (I) Boettiger et al. (2013), (II) Mander et al. (2017), (III) Dakos et al. (2015) and (IV) Dakos et al. (2011). The framework is not necessarily hierarchical [Colour figure can be viewed at http://wileyonlinelibrary.com]

Here, we propose a framework to systematically assess the prerequisites for successful application of SEWS to spatially gridded remotely sensed (‘snapshot’) data from real‐world landscapes. The framework aims to move from an ecosystem approach towards a landscape approach, recognizing that ecosystem properties are partially imposed by geological (lithological) variation, and are affected by human activity. The novel framework (Figure 1) builds on a literature review, supported by spatial analyses and model simulations using examples from real‐world landscapes. The framework is structured to assess three groups of prerequisites for successful application of SEWS to real‐world landscapes. These groups are (1) conceptual, (2) site‐related and (3) data‐related, and are described in the next three sections. Given the focus on practical considerations, our emphasis is on site and data‐related aspects. After outlining the prerequisites, we provide potential solutions and identify key research gaps (Table 1) to further operationalize SEWS theory in real‐world landscapes.

## 2 CONCEPTUAL PREREQUISITES

The following section discusses prerequisites for the use of SEWS and links to Figure 1 by the numbers in the section headings. Given our focus on practical considerations, we briefly review conceptual prerequisites and refer to Boettiger, Ross, and Hastings (2013) and Dakos et al. (2015) for a more detailed overview of conceptual limitations.

### 2.1 Evidence for alternative stable states (1)

Before identifying impending regime shifts or underlying drivers by applying SEWS in for example space‐for‐time approaches, evidence for the potential occurrence of regime shifts, alternative states and hysteresis needs to be established empirically and/or using mechanistic models (Boettiger et al., 2013; Dakos et al., 2015). Alternative states originate from positive feedbacks that maintain the system in a status quo. As a result, the response trajectory of the system state to environmental drivers differs between increasing and decreasing environmental stress, also referred to as hysteresis (Scheffer et al., 2001). Hysteresis is thus a phenomenon that inherently connects to the occurrence of alternative states and regime shifts (Box 1).

The prerequisite of a hysteretic driver‐state relationship has major implications for establishing data‐based evidence of regime shifts using space‐for‐time approaches, where temporal variability in drivers is replaced with variation in space. In practice, evidence of hysteresis requires that the effects of both increasing and decreasing environmental pressure are observable in spatial datasets. Thus, even when nonlinear threshold responses to environmental drivers are observed in real‐world data, such observations not necessarily evidence the existence of alternative states (Phillips, 2003).

For many ecosystems, such as semi‐arid woodlands and savannahs (Rietkerk et al., 2002; Van Langevelde et al., 2003), northern peatlands and marine systems (Hilbert et al., 2000; Möllmann & Diekmann, 2012), climate change is proposed as key driver for regime shifts. Given that temperature is projected to increase worldwide (IPCC, 2013), and because precipitation depends on temperature (Allen & Ingram, 2002), it will be challenging to establish both decreasing and increasing climatic pressure to demonstrate the presence of hysteresis in space‐for‐time approaches.

### 2.2 Effects of regime shift type on the applicability of EWS (2 & 3)

A broad range of mechanisms may result in transitions between ecosystems states, including bifurcations, smooth transitions and rapid regime shifts (Boettiger et al., 2013). Currently, saddle‐node bifurcations have received most attention in research on regime shifts and EWS. Indeed, mathematical analysis demonstrated that critical slowing down (CSD; Box 1) is a universal phenomenon for saddle‐node bifurcations (Wissel, 1984). However, there are many other bifurcation types (van Voorn, Kooi, & Boer, 2010) that do not exhibit CSD or for which it remains unexplored under which conditions CSD occurs (Boettiger et al., 2013) and whether SEWS can be applied. Likewise, CSD may also occur during smooth, nonhysteretic, ecosystem transitions (Hastings & Wysham, 2010; Kéfi, Dakos, Scheffer, Van Nes, & Rietkerk, 2013). Furthermore, sudden extreme changes in drivers, such as periods of extreme drought, may trigger ecosystem regime shifts (Ratajczak et al., 2017) that cannot be predicted with SEWS, since these require slowly changing drivers (Dakos et al., 2015). Accordingly, given the uncertainty associated with other types of regime shifts, a conceptual prerequisite in the application of SEWS is that critical slowing down precedes the specific type of regime shift.

### 2.3 Effect of spatial interaction type on critical slowing down (4)

Critical slowing down may not be present under all types of spatial interactions that result in spatial patterns. For scale‐dependent feedbacks, where vegetation stimulates the performance of itself and its surroundings, but reduces it farther away (due to e.g. resource accumulation, Rietkerk & van de Koppel, 2008), the SEWS in Box 1 fail to indicate upcoming regime shifts (Dakos et al., 2011). Qualitative changes in spatial patterns (Rietkerk, Dekker, de Ruiter, & van de Koppel, 2004) are likely more fruitful indicators of imminent regime shifts in such systems (Dakos et al., 2011; Kéfi et al., 2014).

## 3 SITE PREREQUISITES

### 3.1 Effect of spatially variable environment on spatial patterns (6)

In the previous section we showed that conceptual prerequisites need to be met before SEWS can be calculated from snapshot data. Given the focus on site‐ and data‐related issues, we assume in the following sections that such conceptual prerequisites are satisfactorily fulfilled.

In contrast to homogeneous ecosystems where vegetation patterns arise merely from self‐organizing processes, spatial variability in heterogeneous real‐world landscapes may also arise from spatial variability in environmental conditions. This adds a complication to the application of SEWS, which in theory assume homogeneous landscapes. It is widely accepted that vegetation patterns are related to habitat conditions at, or even within, the plot scale (~1 m^2^) (Cirkel, Witte, van Bodegom, Nijp, & van der Zee, 2014; Ellenberg, 1974; Kohn & Walsh, 1994). Soil texture and chemistry, parent material and lithology, soil depth, (micro)topographic position and soil fauna are examples of factors that control habitat conditions (Cirkel et al., 2014; Dangerfield, McCarthy, & Ellery, 1998; Furley, 1976). These factors, in turn, control plant growth and competition. Thus, spatial vegetation patterns may—in real‐world landscapes—originate both from self‐organizing processes and from heterogeneous environments dictated by topography and geology (lithology) (Bestelmeyer, Ward, & Havstad, 2006; Sheffer, von Hardenberg, Yizhaq, Shachak, & Meron, 2013).

To illustrate how soil‐vegetation relationships may contribute to spatial patterns, we here focus on the Serengeti‐Mara savannah ecosystem (Reed, Anderson, Dempewolf, Metzger, & Serneels, 2008), one of the most extensive savannahs of the world. In savannahs, forest prevails under conditions with high rainfall, low fire frequency and/or low grazing pressure (Hirota, Holmgren, Van Nes, & Scheffer, 2011; Van Langevelde et al., 2003). Adverse changes in these drivers may trigger a regime shift from a forested or savannah state to a homogeneous grassland state (Van Langevelde et al., 2003).

**Figure 2:**
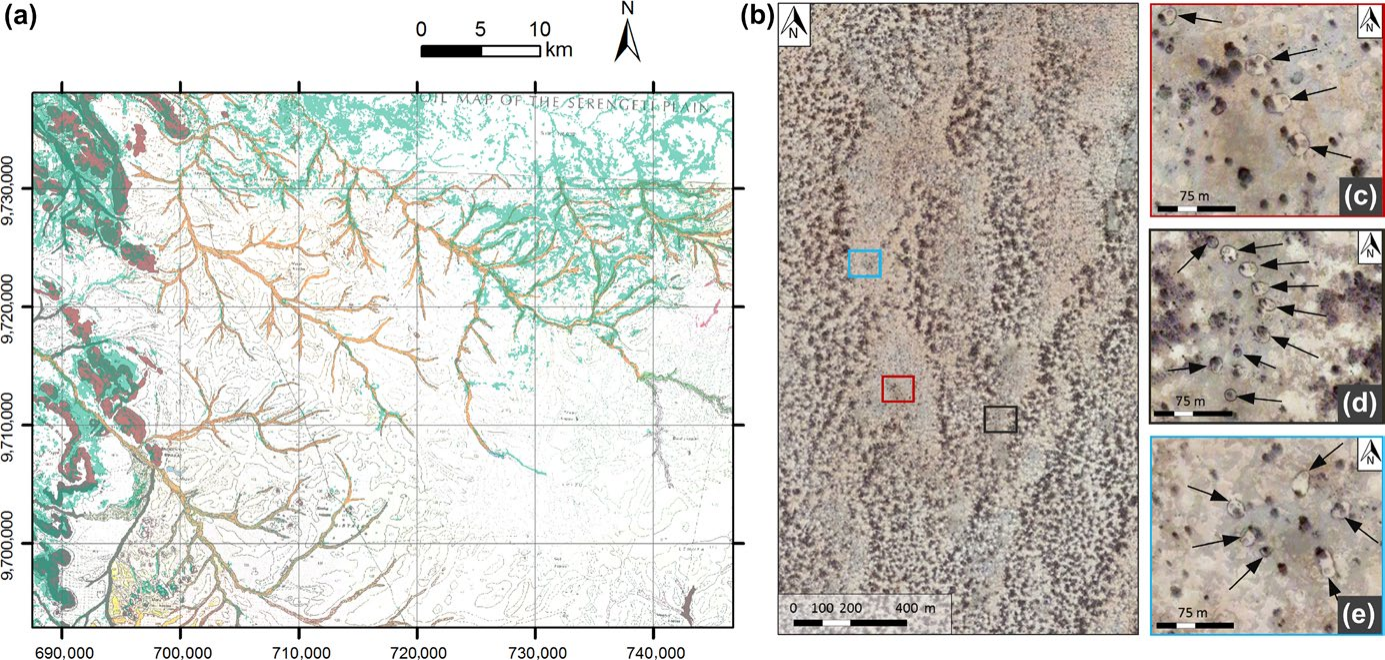
Examples of externally imposed drivers for spatial vegetation patterns. (a) Effect of soil variability on vegetation distribution in the Serengeti‐Mara savannah system (1°47′S 34°33′E). A vegetation map (green = forest, white = grass) is superimposed on a soil map (orange = clayey soils of valley bottoms and riverbeds, red = loamy soils of hills and steep slopes in uplands). The vegetation map is derived from Reed et al. (2008) and classified in forest and grass following Eby et al. (2017); the soil map is modified after de Wit (1978). (b) Semi‐arid woodland in southern Sudan (11°24′N 28°0′E) showing reduced vegetation presence around scattered homesteads (arrows) (c–e) illustrating anthropogenic impact on vegetation patterns (Imagery source: 2 February 2014, Google Earth, DigitalGlobe, 2018) [Colour figure can be viewed at http://wileyonlinelibrary.com]

Based on snapshots from a ground‐truthed vegetation map along rainfall gradients in the Serengeti‐Mara system, Eby et al. (2017) identified a threshold response of grassland cover, which rapidly dropped with increased rainfall. The reduced resilience with increased rainfall was signalled with the SEWS used in Box 1, apparently demonstrating the potential of SEWS in revealing impending regime shifts. However, when comparing vegetation and soil patterns from a digitized soil map (de Wit, 1978), a striking similarity becomes apparent (Figure 2a). Forest presence and patterns appear to be largely constrained to clayey soils in valleys and loamy soils of the uplands. This example illustrates that, even when theory dictates that patterns emerge from self‐organization, environmental heterogeneity imposed by the landscape template not accounted for in drivers may control actual vegetation patterns. Although one may test the correlation between vegetation and geological (lithological) factors, at least two aspects of interpolated global soil data products complicate such correlation tests (see Supporting Information S2).

Ultimately, the relative contribution of spatially variable environmental factors to self‐organizing processes will determine the importance of environmental heterogeneity in affecting spatial vegetation patterns. In case environmental heterogeneity dominates, it will constrain the applicability of SEWS.

### 3.2 Anthropogenic effects on spatial vegetation patterns (7)

Human influence is another source of imposed spatial heterogeneity that may considerably affect SEWS in real‐world landscapes. While certain human influences may be easily identifiable on remotely sensed imagery, other forms are more subtle and may remain unnoticed. An example of such subtle influence arises in semi‐arid woodlands in Sudan (Figure 2b; 11°24′N 28°0′E). Under natural conditions, the land cover of semi‐arid woodlands generally organizes in a distinct two‐phase mosaic of a vegetated state with woody shrubs and a bare soil state (Rietkerk et al., 2002). In semi‐arid woodlands, bistability of a vegetated and a desert state can occur along gradients of rainfall (Rietkerk et al., 2002) and grazing pressure (Noy‐Meir, 1973), and once transformed into desert, a vegetated state may be hard to re‐establish, putting local societies at risk.

At large spatial extents, the semi‐arid vegetation in Sudan appears relatively natural (Figure 2b). Closer inspection, however, reveals distinct areas with low tree abundance surrounding scattered homesteads (Figure 2c–e). In Sudan, deforestation is a major cause of land degradation, where 55% of energy consumption (in 2015) originates from firewood and charcoal (International Energy Agency, 2018) and woodland is converted in agriculture (Aleman, Blarquez, & Staver Carla, 2016). The reduced tree cover surrounding homesteads thus likely resulted from logging for firewood and charcoal, and/or increased herbivory associated with pastoral livestock farming (Figure 2c–e), rather than the homesteads being built preferably in areas of low tree cover. The homesteads are particularly small features (~10 m^2^), and are therefore easily overlooked in large‐scale studies. Nonetheless, human activity can have major impacts on surrounding vegetation patterns, and thus on conclusions drawn from SEWS in socio‐ecological landscapes (Zurlini et al., 2014).

### 3.3 Effects of landscape heterogeneity on regime shift characteristics (5)

Heterogeneity in environmental conditions imposed by the landscape, as discussed above, can affect vegetation and needs to be considered before interpreting patterning in the framework of SEWS and regime shifts. This section discusses two pathways through which landscape heterogeneity affects ecosystem resilience and properties of regime shifts at the landscape level.

#### 3.3.1 Effects of spatially varying growing conditions on landscape‐level regime shifts

As shown in the previous section, vegetation cover may vary as a function of landscape position and environmental characteristics. Such spatial variability of environmental factors leads to spatial differences of vegetation to survive or recover from disturbance (Ratajczak et al., 2017). At the landscape level, this implies that the response to gradually increasing spatially averaged stress is no longer instantaneous in parameter space. Instead, due to spatial heterogeneity, vegetation at different landscape positions will fail at different levels of external stress. Overall, this yields a more gradual response to environmental changes, as for instance illustrated by van Nes and Scheffer (2005) using one‐dimensional model experiments describing grazing pressure effects on vegetation growth (Noy‐Meir, 1975).

In the model experiments by van Nes and Scheffer, gradual and random spatial heterogeneity affected vegetation response to increasing grazing pressure, but not vegetation recovery from decreasing grazing pressure. Because vegetation recovery remained sudden, this resulted in substantial hysteresis even in systems with spatially varying growing conditions. However, later modelling work by Martín, Bonachela, Levin, and Muñoz (2015), who explored the effect of stochasticity, dispersion and spatial heterogeneity on ecosystem regime shifts, confirmed that also landscape‐level recovery should be expected to be more gradual and without hysteresis under realistic assumptions. Moreover, spatially heterogeneous diffusion may lead to stable co‐existence of alternative states in spatially extended heterogeneous systems (van de Leemput, van Nes, & Scheffer, 2015). These modelling studies raised new questions about the existence of multiple stable states and regime shifts in, and at the scale of, real‐world landscapes.

#### 3.3.2 Impact of connectivity on regime shift characteristics

Topographic and/or lithological heterogeneity in a real‐world landscape promotes redistribution of resources between locations. A good example of this is redistribution of water from hillslopes to valleys (Puigdefabregas, Sole, Gutierrez, Del Barrio, & Boer, 1999). With increasing drought, reduced plant density at drier upslope positions may increase the water availability at downslope landscape positions (Puigdefábregas, 2005). Plants in these receiving positions will be better able to survive stress than they were before—a spatially explicit case of scale‐dependent feedbacks following, for example Rietkerk et al. (2002). The gain for plants in receiving landscape positions makes these ‘oasis’ positions function as refugia, where plants can survive harsh conditions and from where they can recolonize landscapes if conditions improve (Trichon, Hiernaux, Walcker, & Mougin, 2018).

Clearly, redistribution of water requires hydrological connectivity between landscape positions. In some landscapes, this requirement may not be met, such as very sandy semi‐arid landscapes where all water can infiltrate and no overland flow or upward seepage occurs, or karst landscapes with many small depressions. However, most landscapes do appear to have substantial connectivity, at least during intense rainfall (Okin et al., 2015).

**Figure 3:**
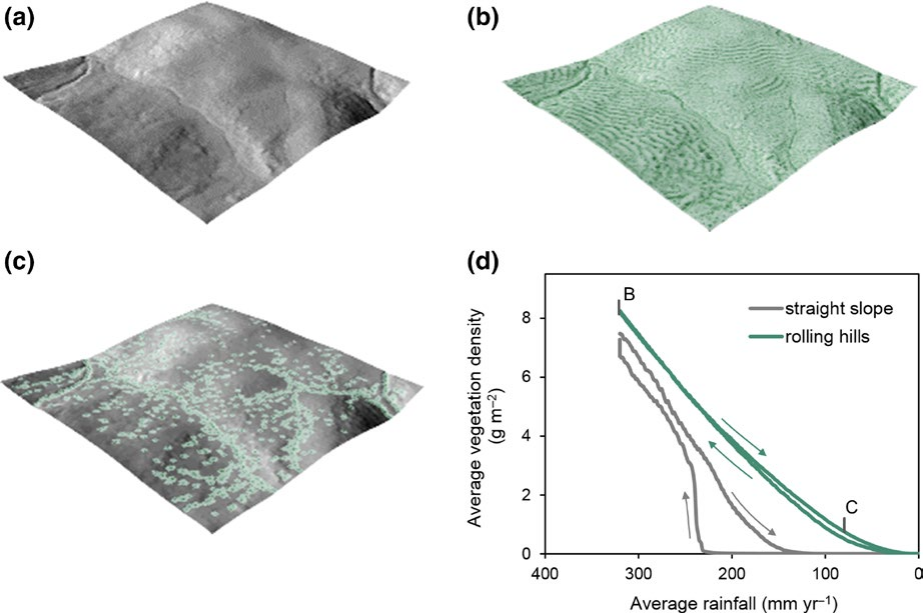
Modelled effects of landscape heterogeneity on vegetation recovery from changing environmental drivers and regime shift characteristics (a) A hydrologically connected landscape with rolling hills. The landscape extent is 1 km^2^ and cell size is 25 m^2^. Total relief is 16 m. (b) Simulated banded vegetation pattern under semi‐arid conditions (320 mm/year, see Fig. d). Vegetation density ranges from 64 to 0 g/m^2^. (c) Persistence of vegetation under arid conditions (75 mm/year, see Fig. d) in positions that receive run‐on water flow. Vegetation density ranges from 64 to 0 g/m^2^. (d) Landscape‐averaged response and recovery curves indicating extremely limited hysteresis and very gradual response for the rolling hills landscape. The grey line shows a reference result for a straight slope landscape, with sudden recovery at rainfall 225 mm/year, leading to substantial hysteresis. Arrows indicate directions of ecosystem response trajectories to rainfall [Colour figure can be viewed at http://wileyonlinelibrary.com]

To illustrate the impact of gradually increasing stress (drought) on such ecosystems in heterogeneous landscapes, we compared vegetation response to drought on a homogeneous straight slope and a rolling landscape in California, US (Figure 3a) using a model that accommodates for both vegetation and landscape dynamics (see Baartman, Temme, and Saco (2018) for model details and parameterization). The model results confirm the hypothesis that retreat of vegetation from drier, upstream landscape positions allows vegetation in downslope, receiving positions to survive longer than it would in a flat, nonheterogeneous landscape (Figure 3). At the ecosystem level, this implies that both response and recovery would be more gradual in landscapes with topographic/geologic heterogeneity than more simple landscapes without such hydrological refugia (Figure 3d). These model results are in line with observations of a semi‐arid grass species response to drought by Godfree et al. (2011), who found that even small topographic variability (0.2–3 m) was sufficient to sustain grass cover after drought.

In summary, heterogeneity and connectivity at the landscape scale seemingly work in the direction of more gradual ecosystem responses to and recovery from stress, hence promoting resilience at the ecosystem level. It remains to be addressed to what extent such landscape‐level resilience affects the applicability of SEWS.

## 4 DATA‐RELATED PREREQUISITES

### 4.1 Categorical data effects on spatial EWS (8)

Two main approaches are adopted to translate a remotely sensed signal into indicators of ecosystem status: land cover classification (categorical data) and the calculation of vegetation indices (continuous data) (Xie, Sha, & Yu, 2008). Both categorical and continuous spatial data are used to calculate SEWS (Kéfi et al., 2014).

**Figure 4:**
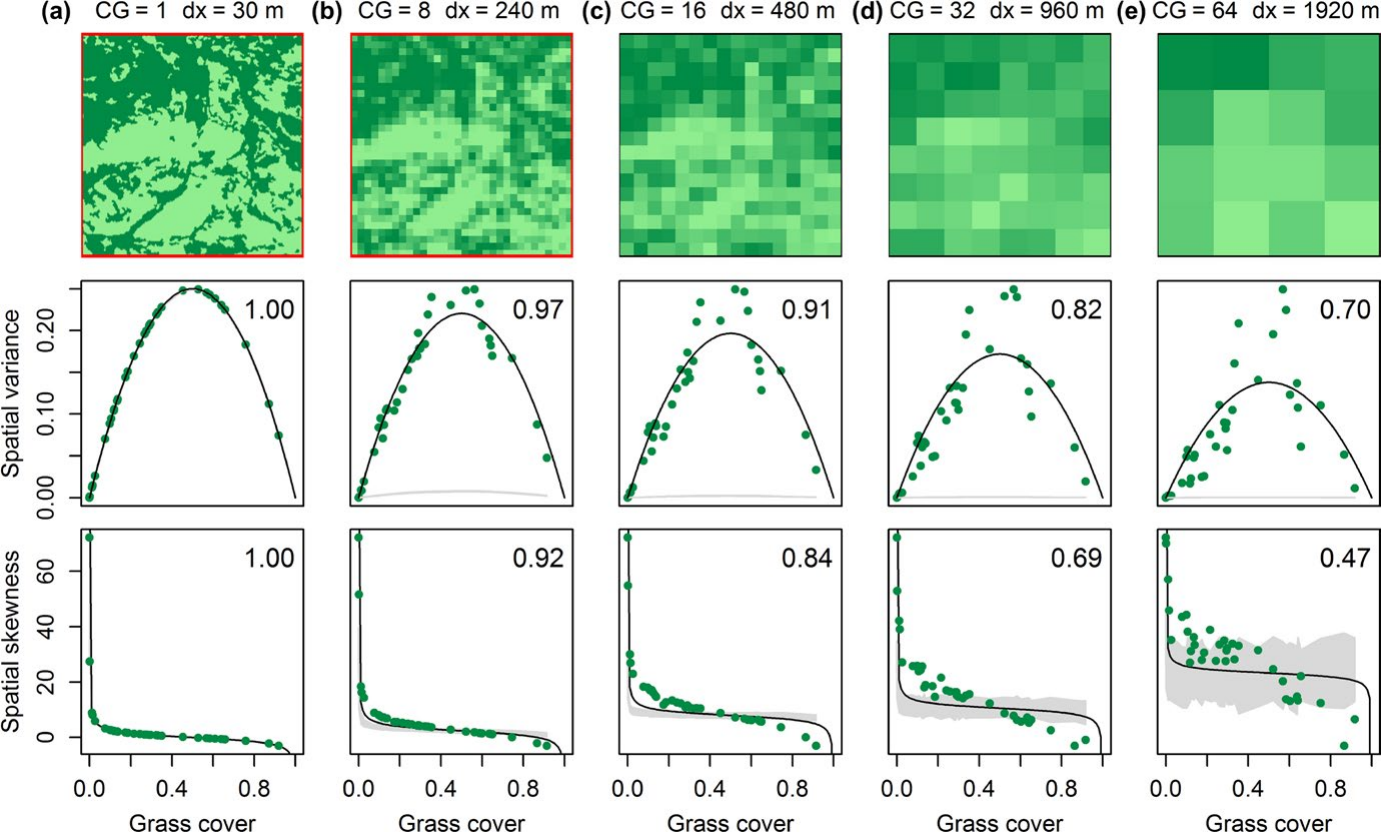
Dependence of spatial early warning signals on mean field value persists with coarse‐graining. The top row illustrates the effect of coarse‐graining (increasing with columns a to e) on spatial patterns of grassland (bright colours) and forest (dark colours) in a representative spatial snapshot (7.5 × 7.5 km) of the Serengeti‐Mara ecosystem. CG denotes the coarse‐graining factor and dx the grid resolution, red or black borders indicate bimodal or unimodal distribution of grass cover. The middle and bottom row show suggested SEWS spatial variance and skewness as a function of mean grass cover and how they depend on coarse‐graining pre‐processing. Snapshot data are taken along a rainfall gradient. Numbers in top‐right of each graph indicate the goodness of fit (*R* ^2^_adj_) of the theoretical mean versus variance or skewness relationships, which were all significant (*p* < 0.05). Grey areas correspond to 95% confidence intervals of early warning signals acquired from 200 simulated null‐models (through random permutation snapshot grid cells conform Kéfi et al., 2014) [Colour figure can be viewed at http://wileyonlinelibrary.com]

For categorical vegetation maps, SEWS are calculated from presence–absence data. This ‘binarization’ introduces challenges in the calculation of the SEWS spatial skewness and spatial variance. Both skewness and variance are mathematically directly dependent on the mean field value for binary data and therefore not only dependent on spatial patterns (Sankaran, Majumder, Kéfi, & Guttal, 2017). This theoretical relationship was confirmed in our analyses of grass cover along a rainfall transect in the Serengeti‐Mara system (Figure 4a). The changes in spatial variance and skewness may thus not exclusively reflect modifications in grass cover patterns, but also a modified mean cover. This may lead to false and misleading regime shift identification.

To circumvent the dependence on the mean, categorical data can be transformed into semi‐continuous maps using coarse‐graining (Génin, Majumder, Sankaran, Schneider et al., 2018). Coarse‐graining is a pre‐processing step that aggregates grids to a coarser resolution, where the aggregated cell values represent mean values of the binary data. The required degree of coarse‐graining is estimated as the aggregation level at which the originally binary input data shifts to a unimodal distribution (Sankaran et al., 2017). We tested multimodality in grass cover distribution of the Serengeti‐Mara dataset at various coarse‐graining levels using Hartigan dip tests (Hartigan & Hartigan, 1985; R Core Team, 2018).

Despite coarse‐graining, however, the strong relationship between mean and spatial variance/skewness persisted (Figure 4b–c), even when the original bimodal distribution was modified to a unimodal distribution and spatial patterns were removed. This illustrates that these SEWS largely originate from mean state changes along the transect rather than a modified spatial pattern, even at high coarse‐graining levels. In addition, as spatial patterns vary both in space and time, the optimal coarse‐graining level required to establish a unimodal distribution would vary spatiotemporally, which further complicates SEWS analysis. These unfavourable properties illustrate that application of spatial variance and spatial skewness as indicators of regime shifts to categorical data is delicate at best.

Furthermore, for both categorical and continuous data, the specific dataset used for SEWS analysis and preprocessing techniques needs to be carefully selected. Not only pattern characteristics (see Supporting Information S4 for an example), but even the number of alternative stable states may vary with data product specifications (Xu et al., 2015). In systems with substantial seasonality, vegetation activity and its spatial pattern will also depend on acquisition time (Rasmussen et al., 2011).

### 4.2 Impact of definition and number of ecosystem states (9 + 10)

Besides the data‐related issues described above, the process of defining ecosystem states based on remotely sensed imagery may be highly challenging. Continuous maps of vegetation indices derived from remotely sensed imagery (e.g. colour or NDVI) allow for gradual changes of states in space, but do not provide a direct characterization of vegetation types or ecosystem states. Threshold values are needed to distinguish between vegetation types, but values of vegetation indices often overlap for different vegetation types, and cut‐off values may even be subjective (Xie et al., 2008; Yan, Wang, Lin, Xia, & Sun, 2015), leading to ambiguous ecosystem state definitions. Yet, for some SEWS, such as those based on pattern morphology (Mander et al., 2017) or changes in patch‐size distributions (Kéfi et al., 2007), classification of continuous data into distinct classes is an inevitable preprocessing step.

#### 4.2.1 Effect of (not) merging discrete land cover types in ecosystem states (10)

**Figure 5:**
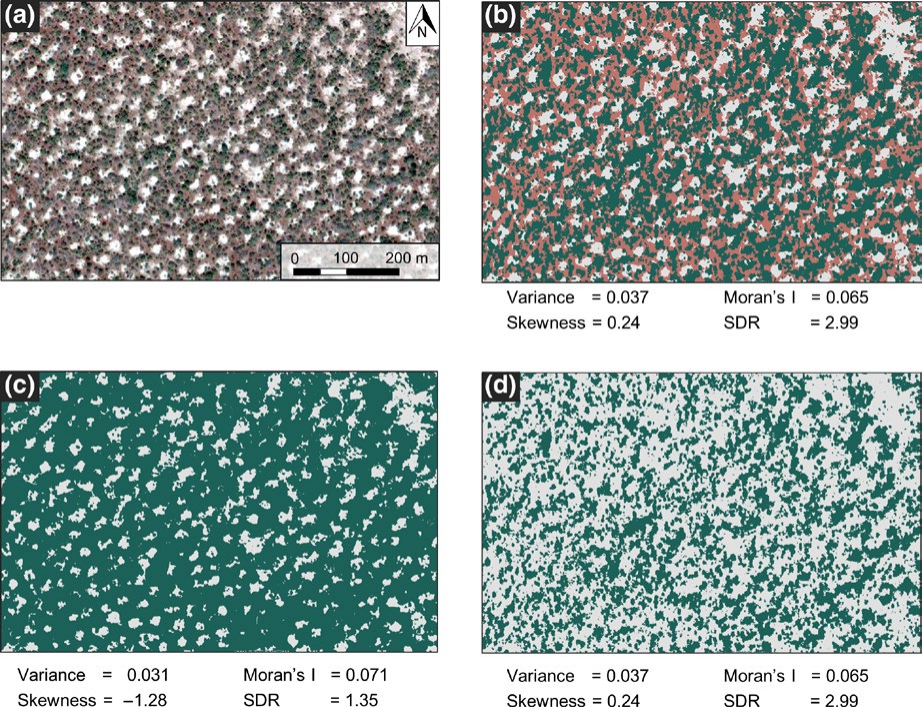
Effect of alternative stable state definitions on spatial early warning signals (a) Vegetation pattern of semi‐arid woodland in the Sahel region in Sudan (11°7′N 28°15E, image source: 11 September 2014, Google Earth © 2018, Digital Globe 2018) showing three distinct colours that are associated with vegetated (green), bare (white) and ‘herbaceous plants’ (brown‐red) cover. (b) Classified vegetation map with three land cover types. (c) Classified vegetation map where the red land cover type is merged with the ‘vegetated’ state or (d) with the bare soil state. Early warning signals (numbers below figures) are calculated for the forested state [Colour figure can be viewed at http://wileyonlinelibrary.com]

Alternative states may be characterized by a range of internal variation, which makes it difficult to identify alternative stable states in snapshots. For instance, in semi‐arid woodlands in Sudan (11°7′N 28°15′E), three land cover types occur with clearly distinct spectral properties (Figure 5a). Whereas vegetation of such semi‐arid systems is typically classified in ‘bare soil’ (whitish colour) and ‘vegetated’ (greenish) (e.g. Rietkerk et al., 2002), about 33% of the total area in the example is represented by a third land cover type with a distinct reddish colour (Figure 5a; cover determined with supervised maximum likelihood classification, see Supporting Information S3). Although no ground observations are available for confirmation, the red colour likely characterizes ‘herbaceous plants’ (Barbier, Couteron, Lejoly, Deblauwe, & Lejeune, 2006), representing a distinct biomass group between bare soil and forest. The same three‐phase mosaic is observed in many other parts of the Sahel region, including for example Chad (12°9′N 17°36′E), Senegal (14°25′N 14°30′W) and Mali (14°9′N 6°44′W) on Digital Globe imagery using Google Earth (Google, CA). The aggregation of this ‘herbaceous’ state with the vegetated or bare soil state significantly affects vegetation patterns (Figure 5a) and cover of the vegetated state (47% vs. 80%) in the Sudan example. As a result the SEWS also vary considerably with state definitions (Figure 5). The difference in SEWS between merging the red ‘state’ to the forested state and merging it to the bare state is well above 100% for spatial skewness and spectral density ratio (Figure 5c–d). Therefore, erroneously merging land cover types may result in failure of timely regime shift detection.

Alternatively, the herbaceous plant community may represent a third alternative stable state (Figure 5b), as also proposed by Holmgren and Scheffer (2001) for arid ecosystems and van Nes, Hirota, Holmgren, and Scheffer (2013) for tri‐stable tropical tree cover. Considering the red land cover as a separate regime affects the cover and spatial patterns of the forest and bare soil states, hence SEWS. Hypothetically, disaggregating two alternative stable equilibria into multiple (>2) equilibria may reduce the impact of regime shifts, as tipping to an intermediate state may have a smaller impact on ecosystem state, and the irreversibility (hysteresis domain) is likely to reduce with increasing number of stable states.

In the examples above, different criteria to merge land cover types will result in different spatial patterns. Comparable issues with defining ecosystem states may appear in other patterned ecosystems around the world, for example, in northern peatlands (Korrensalo et al., 2018). The—often subjective—choices for merging states influence spatial patterns, SEWS and ultimately the estimated likelihood of regime shifts.

### 4.3 Selecting the right image resolution (11)

The suitability of remotely sensed imagery for describing patterns, hence usage in SEWS analyses, crucially depends on its resolution and the spatial ecosystem pattern of interest (Woodcock, Strahler, & Jupp, 1988). If the image resolution is coarser than the typical vegetation pattern, a remotely sensed signal may be composed of multiple vegetation types. Such spectral mixing due to coarse resolution generally reduces classification accuracy (Roth, Roberts, Dennison, Peterson, & Alonzo, 2015), and has major impact on ecosystem pattern characteristics and proportional cover of vegetation classes (Moody & Woodcock, 1995; Wickham, O'neill, Riitters, Wade, & Jones, 1997) (Figure 4). At fine resolutions, the classification accuracy may be reduced due to increased within‐class variability due to for example variation in illumination and shadow (Löw & Duveiller, 2014; McCloy & Bøcher, 2007), which will affect spatial patterns and therefore SEWS. Thus, both too coarse and too fine image resolution may affect SEWS and conclusions drawn.

Typically, remote sensing products with a longer history (e.g. Landsat or MODIS) have coarser resolution than more recent imagery (e.g. Sentinel‐2). This implies that the required resolution will determine whether longer‐term datasets are available to reconstruct historical developments in SEWS and upcoming regime shifts over time.

## 5 OPPORTUNITIES AND FUTURE DIRECTIONS

### 5.1 Confronting conceptual challenges

Currently, not all types of impending regime shifts can be identified with SEWS due to for example limited applicability of critical slowing down (CSD) or occurrence of extreme events that are impossible to anticipate using contemporary SEWS (Boettiger et al., 2013; Dakos et al., 2015). Major research efforts are aimed to estimate the likelihood of environmental extremes using mechanistic models (Fischer & Knutti, 2014; IPCC, 2013; Nijp et al., 2017) and controlled experiments (Knapp et al., 2008; Nijp et al., 2014). Such studies are crucial to identify when the duration or intensity of environmental extremes is sufficient to trigger irreversible regime shifts, and hence may demarcate quantitatively under which driver changes SEWS may be useful indicators for regime shifts (Table 1).

For space‐for‐time approaches at large spatial extent with climate as driver, it is challenging to demonstrate hysteresis, as both a cooling and warming climate are required but the vast majority of the earth is warming (IPCC, 2013). A solution to establish the particularly elusive ‘cooling climate branch’ may lie in historical point‐data, such as paleo‐ecological records (Cole, Bhagwat Shonil, & Willis Katherine, 2015; Davies, Streeter, Lawson, Roucoux, & Hiles, 2018; Willis, Bailey, Bhagwat, & Birks, 2010). However, such point‐data might not reflect the spatiotemporal evolution of spatial patterns. Interesting opportunities for successful resilience assessment may arise by combining research methods (particularly paleo‐ecology, mechanistic models, theoretical concepts, remotely sensed data and plant trait databases). For instance, Spasojevic et al. (2015) successfully combined remote sensing with trait datasets to gain insight in ecosystem recovery from wildland fire, and Rogers et al. (2018) combined tree growth observations with remote sensing to successfully detect regime shifts with temporal EWS. These recent examples illustrate how integration of research methods can lead to novel insights in the application of SEWS in real, heterogeneous landscapes.

### 5.2 Resolving issues related to landscape heterogeneity

Real‐world ecosystems are often affected by socio‐ecological interactions that raise substantial environmental heterogeneity (Figure 2). Model simulations indicate that geomorphic landscape heterogeneity likely reduces hysteresis, results in a smoother, more linear driver‐state relation, and due to landscape refuges allows vegetation to persist under environmental change (Figure 3; Martín et al., 2015; van Nes & Scheffer, 2005). These model results are confirmed by observations in semi‐arid woodlands in the Sahel (Trichon et al., 2018) and a perennial grassland (Godfree et al., 2011), which demonstrate the importance of heterogeneity for landscape‐level resilience. Nevertheless, current theoretical and empirical understanding of how spatially structured heterogeneity affects SEWS is highly limited (but see Génin, Majumder, Sankaran, Schneider et al., 2018 for spatially variable stressors caused by autogenous processes). Filling this research gap is crucial for the detection of impending regime shifts in real‐world socio‐ecological systems, and requires moving from an ecosystem approach towards a landscape perspective (Table 1).

The heterogeneity of environmental factors may control spatial vegetation patterns and interfere with SEWS and regime shifts detection (Figure 2). Before the application of SEWS, it needs to be confirmed that state variables are not disproportionally associated with spatially variable environmental conditions or societal actors (e.g. soil type, logging) using chi‐square tests or cross‐variograms (Rossi, Mulla, Journel, & Franz, 1992). Unfortunately, global spatial data on, for example, soils (Hengl et al., 2017; Stoorvogel, Bakkenes, Temme, Batjes, & Brink, 2016) is often highly uncertain at a resolution matching the typical scale of vegetation patches (~1–250 m; Supporting Information S2). A prospective high‐resolution alternative for soil and geology data may be found in digital terrain metrics (e.g. topographic wetness index, slope, curvature; Wilson, 2012), derived from remotely sensed digital elevation information. Geology and topography are important controls on soil‐forming processes and water availability (Jenny, 1941), and as such may serve as proxy for soil properties (Moore, Gessler, Nielsen, & Peterson, 1993).

Besides verifying controls on spatial patterns, environmental datasets may be used as alternative null‐models. In state‐of‐the‐art SEWS analyses, spatial patterns on snapshots are compared to null‐models representing random noise (Génin, Majumder, Sankaran, Schneider et al., 2018; Kéfi et al., 2014). These null‐models allow for testing the hypothesis that vegetation structure is significantly different from random noise. Instead, by taking spatial patterns of environmental variables as null‐models, it can be tested whether spatial vegetation patterns and SEWS are significantly different from spatial variability in the environment. Such null‐models have not yet been developed, but would considerably advance SEWS application (Table 1).

The effect of socio‐economic activities (e.g. logging) on spatial vegetation patterns cannot be ruled out in the Anthropocene (Figure 2), and its importance requires careful examination before the application of SEWS. In many data‐sparse regions considerable socio‐economic activity occurs, while the exact human impact on vegetation patterns remains largely undocumented (Chidumayo & Gumbo, 2013). In such conditions, object based image analyses, which utilizes information of texture, shape, and context, may become useful to detect human structures (Blaschke et al., 2014).

### 5.3 Resolving data‐related issues

**Figure 6:**
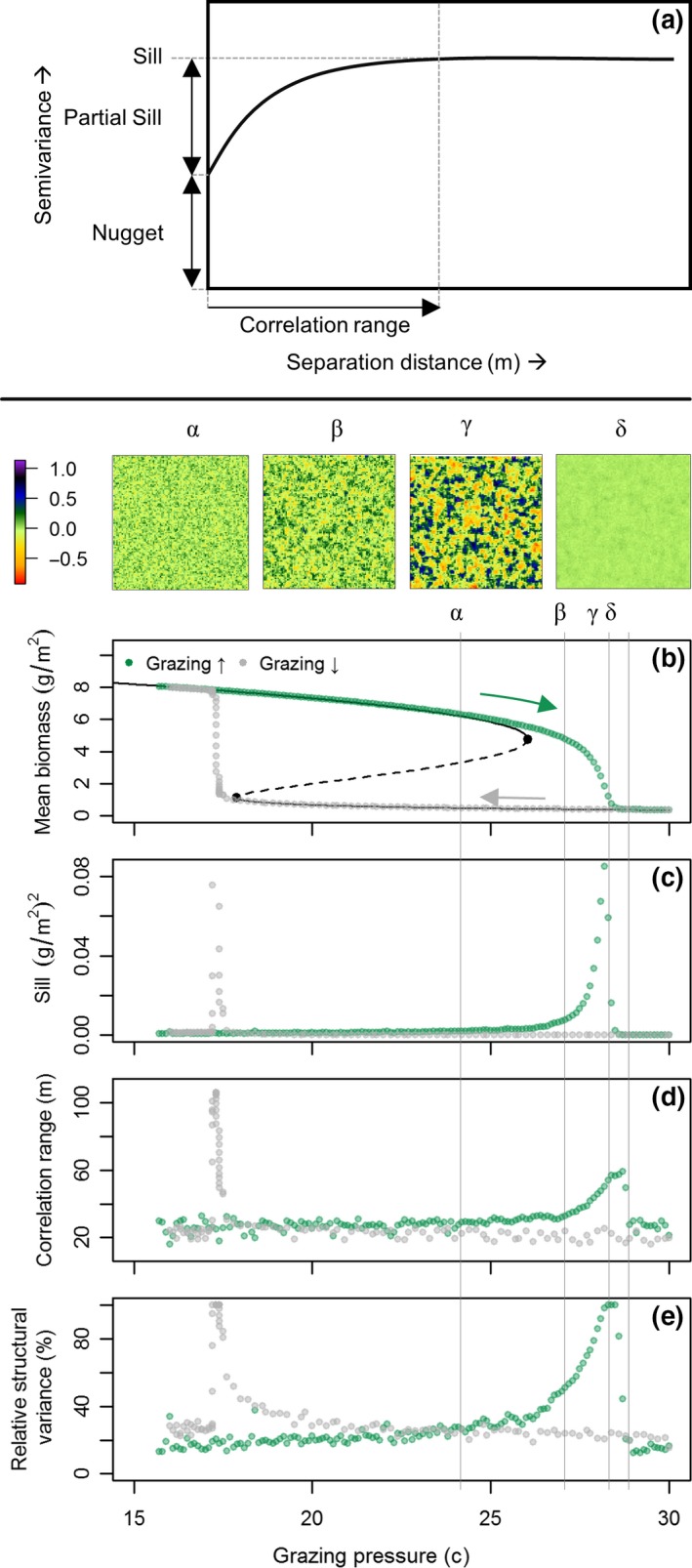
Variogram analyses as prospective spatial early warning signal. (a) Definition of variogram parameters that characterize spatial heterogeneity. The *x*‐axis represents the separation distance between points in space, the *y*‐axis the mean variance at this separation distance averaged over multiple point‐pairs (referred to as semivariance). Typically, the similarity decreases with increasing separation distance, increasing the semivariance. The correlation range represents the separation distance up to which points in space are correlated, and can be interpreted as a quantitative measure of mean patch size. The partial sill is the part of variance that is spatially structured, whereas the nugget effect represents a random component. Together they constitute the sill. A variogram consisting of only a nugget represent pure spatial random noise. (b) Vegetation biomass as function of grazing pressure. (c) Variability of biomass in space (sill; nugget + partial sill parameter). (d) Mean patch size (m) of biomass (correlation range parameter): represents the characteristic length scale at which spatial patterns emerge. (e) The relative structural variance (100% partial sill/(partial sill + nugget)), quantifying whether data are spatially structured (100%) or organized randomly (0%) [Colour figure can be viewed at http://wileyonlinelibrary.com]

The use of categorical data introduces several challenges, ranging from reducing the applicability of spatial skewness and variance to difficulties with defining ecosystem states (Figures 4 and 5). When using categorical snapshots, it is crucial to clearly define merging criteria of land cover types in ecosystem states or test the sensitivity of results to other relevant state definitions. Extensive field‐based ecosystem knowledge will therefore remain indispensable for adequate state definitions. Given these issues, we advise to avoid these SEWS with categorical data and focus on alternative SEWS (Berdugo et al., 2017; Dai et al., 2013; Kéfi et al., 2014; Mander et al., 2017; Yin, Dekker, Rietkerk, van den Hurk, & Dijkstra, 2016). Spatial correlation length, another measure for CSD (Dakos et al., 2010), has a strong theoretical foundation in geostatistics and can be quantified from variogram analysis (Journel & Huijbregts, 1978). Variograms (Figure 6) provide a measurable, interpretable and intuitive measure of vegetation patch‐size distribution (Li & Reynolds, 1995; Woodcock et al., 1988), are applicable to binary data, quantify spatial correlation (in random and spatially structured components) and can account for anisotropy (e.g. linear features on sloping terrain) in a single analysis (Rossi et al., 1992; Webster & Oliver, 2007). Additionally, variograms do not, in contrast to wavelength analyses (e.g. Fast Fourier Transforms; Mugglestone and Renshaw (1998)), require equidistant sampling and preprocessing to deal with no data values.

The potential of variograms as SEWS can be illustrated from spatial patterns generated with the Noy‐Meir model (Box 1). The variability, mean patch size and spatial structure all clearly increase with increased grazing pressure (Figure 6; see Supporting Information S5 for analyses details). Though explorative, this analysis demonstrates that variograms very well capture changes in spatial patterns at reduced ecosystem resilience. Given the advantages of variogram analyses, its application to detect impending regime shifts induced by global change deserves further attention (Table 1).

As discussed, the resolution of openly available global remotely sensed products has increased steadily over the past decades and will likely increase further in the future (Toth & Jóźków, 2016). The higher resolution, however, introduces new challenges as it increases within‐class variability, increasing the potential for misclassification and inaccuracies in spatial patterns (Löw & Duveiller, 2014). To estimate the optimal image resolution for SEWS analyses, or to select among appropriate remote sensing products, variograms again become useful. For gridded data, Garrigues, Allard, Baret, and Weiss (2006) recommend to calculate the optimal resolution using the typical length scale of landscapes (*D_c_*), derived from the range parameter of theoretical variograms. With the Shannon Theorem stating that the sampling frequency needs to be higher than twice the maximal frequency of the signal (Shannon, 1949), the optimal resolution thus equals *D_c_*/2.

**Table 1:** Key research priorities in the field of spatial early warning signals (SEWS)

How extreme must single extreme events be to trigger regime shifts?
Can SEWS be used to detect impending regime shifts in heterogeneous landscapes?
Can we develop null‐models to test the hypothesis that vegetation patterns are significantly different from environmental patterns?
How can controls on spatial patterns be identified in data‐sparse regions without information on environmental heterogeneity?
Can variograms provide valuable SEWS (correlation length, part of explained variance valuable), as alternative for spatial skewness and spatial variance?

## 6 CONCLUSION

Real‐world landscapes are complex due to spatial heterogeneity induced by geo(morpho)logical heterogeneity and socio‐ecological interactions. This may lead to several complications when applying SEWS in real‐world landscapes (Figure 1). The first main complication is that spatial environmental variability imposed by landscape template and socio‐economic factors may affect spatial patterns. As SEWS are derived from spatial patterns, such environmental heterogeneity may result in misleading interpretations of regime shifts. Second, heterogeneity in real‐world landscapes may promote landscape‐level resilience, and enhance the reversibility of regime shifts. Moreover, particularly the application of SEWS to categorical maps requires careful consideration of ecosystem state definitions and the specific SEWS employed. For categorical data, spatial variance and skewness may be inappropriate SEWS and variogram analyses, a common geostatistical technique to quantify spatial patterns, seems a fruitful alternative. These and other complications are detailed in the presented framework, as well as opportunities to solve them.

Moving from an ecosystem towards a landscape approach, we hope the framework (1) enhances successful application of SEWS in real‐world landscapes, (2) prevents over‐interpretation of SEWS, (3) provides guidance in understanding relations between spatial vegetation patterns and environmental drivers in landscapes under pressure and (4) will stimulate further development of SEWS that can deal with the challenges outlined in this paper.
