# Supplementary material for: Spatial early warning signals for impending regime shifts: A practical framework for application in real‐world landscapes
Source: Glob Chang Biol. 2019 Apr 1;25(6):1905–21. doi: 10.1111/gcb.14591 (PMC6849843; doi:10.1111/gcb.14591)
Supplement: Supplementary file 1 [file GCB-25-1905-s001.pdf]

S1: Model implementation for biomass regime shifts induced by grazing pressure

To illustrate the functioning of critical slowing down, we employed the Noy-Meir model (Noy-Meir, 1973) that includes spatially explicit grazing that varies in space and time following the implementation of Guttal and Jayaprakash (2009) (Eq. 3). We employed a grid consisting of 129 × 129 cells of 0.1 spatial units (each grid cell representing 100 m^2), with periodic boundary conditions using Euler forward integration with a time step of 0.001 (representing 0.365 days, so that 1000 time steps represent 1 year). All model parameters are similar to those employed by Guttal and Jayaprakash (2009). To simulate the regime shift response of vegetation to increasing grazing pressure, we gradually increased the grazing pressure c with 0.1 every 1000 iterations. To verify the regime shift type of response and test the occurrence of hysteresis, we also employed a scenario with decreased grazing pressure with the same rate of grazing pressure reduction.

S2: On testing spatial pattern controls

To determine the mechanism controlling spatial vegetation patterns, one may test the correlation between vegetation and, for instance, soil. However, two aspects of interpolated global soil data products complicate such correlation tests. First, global soil data products are highly uncertain in regions where underlying data density is low, e.g. in wetlands, semi-arid regions, or the tropics (Hengl et al., 2017). Even at resolution of 250-5000 m, current global soil data products therefore cannot be used to link soil and vegetation patterns, given that the scale of spatial vegetation patterning generally emerges at much smaller scales. Second, global soil data products are often based on remotely-sensed covariates relating to vegetation, such as NDVI (Hengl et al., 2017). This introduces circularity in correlation tests. Digitized local soil maps will therefore often be the only source of information of sufficient detail, but are highly restricted in spatial extent. In many cases, it may thus be hard to disentangle the controls on spatial vegetation patterns, hence determine the credibility of SEWS.

S3: Classification of vegetation patterns in Sudan

The true-colour Digital Globe satellite imagery of semi-arid vegetation in Sudan (Figure 2A in main text) was obtained through the Google Earth Engine. The satellite image was classified into a ‘vegetated’, ‘bare’ and ‘herbaceous’ state using supervised Maximum Likelihood Classification (ArcGIS v10.4). The classifier was trained by manually identifying ten points per class on the image. Sampling point locations were selected based on homogeneous and representative colour. Spectral information was extracted for all pixels within a buffer of 10 meter radius around each point.

The quality of the classified vegetation map was verified through comparing the major predicted vegetation class within a buffer around ten additional points per class. The classification accuracy was assessed with a confusion matrix, which suggested that the classification accuracy was 100% for all three classes. The high accuracy was supported by visual comparison of spatial vegetation patterns among observed and classified images.

S4: Effect of rainfall data source on detecting impending regime shifts

Rainfall is suggested to be an important driver for regime shifts for a broad range of ecosystems, including e.g. savannahs, northern peatlands and semi-arid woodlands (Hilbert, Roulet, & Moore, 2000; Hirota, Holmgren, Van Nes, & Scheffer, 2011; Rietkerk et al., 2002). For a large part of the world, time series of satellite-based observations are available at relatively high spatial resolution since the Tropical Rainfall Measuring Mission was launched (0.25° × 0.25°, TRMM; Huffman et al. (2007)). Such data have huge potential to detect impending regime shifts caused by climate change induced rainfall modifications at large spatial extent. To determine the importance of the exact dataset on detecting impending regime shifts, we compared spatial early warning signals (EWS) along a rainfall gradient in the Serengeti-Mara ecosystem using the broadly available satellite-based TRMM rainfall estimates with a ground-based dataset.

Spatial EWS were calculated conform Kéfi et al. (2014), based on 40 snapshots (2.5 × 2.5 km) of forest-grass patterns (Reed et al., 2008) along a rainfall gradient (transect 5 in Eby et al. (2017)). The mean annual rainfall map based on rain-gauge observations (provided by Dr. M. Coughenour) was created with a Gaussian inverse distance weighing algorithm for interpolation that accounts for spatial variation in altitude (Coughenour, 1992; Eby et al., 2017). The TRMM rainfall data comes at a resolution 0.25° × 0.25°, roughly representing 29 ×29 km. The rainfall transects in the Serengeti-Mara national park with a maximum length of ~100 km, however, cover maximally about 4 gridcells, not allowing for detection of gradual grass cover responses to rainfall. We therefore interpolated rainfall at a finer resolution (2.6 km) using variograms, a common method for spatial interpolation in geostatistics (Webster & Oliver, 2007). The spatial structure of rainfall was parameterized using the INTAMAP package in R (R Core Team, 2018), accounting for anisotropy in spatial rainfall (Fig. S3.1). The TRMM based rainfall is substantially larger than the on-site rainfall estimates, probably caused by the different periods covered (satellite-based: 1998 – 2012; rain-gauge interpolated map: 1960 – 2004). Nevertheless, the spatial rainfall distribution matches well among the data products (linear regression, $R^2_{adj} = 0.75$; $P < 0.001$).

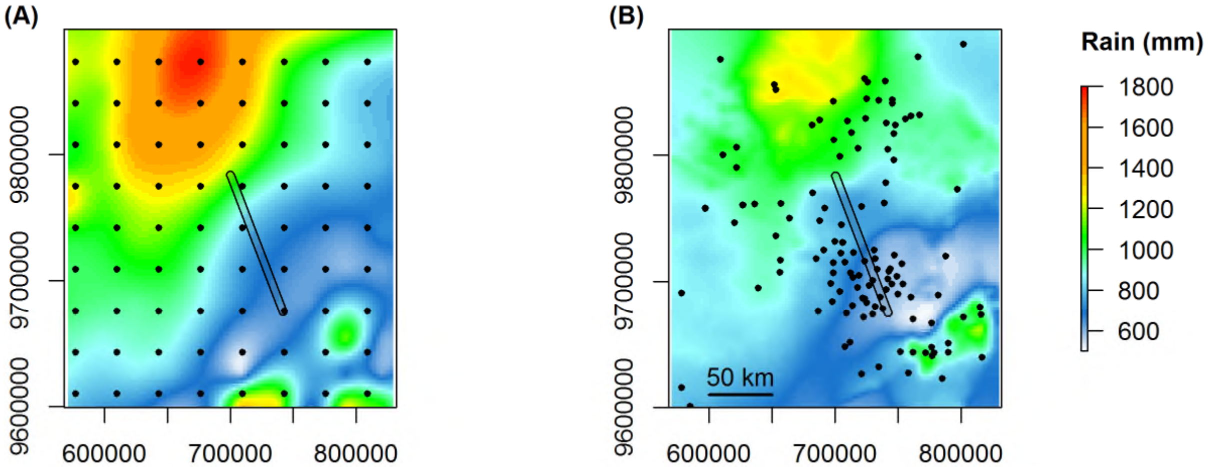

Figure S4.1: Spatial distribution of annual rainfall sum (mm) in the Serengeti-Mara woodland. (A) *Interpolated rainfall based on TRMM satellite data (points) for the years 1998 – 2012. Points represent cell centres of original TRMM observations.* ***(B)*** *Interpolated rainfall (1960 – 2004) based on rain gauge observations (points), accounting for elevation as described in Eby et al. (2017) and Coughenour (1992) (provided by Dr. M. Coughenour). The polygon shows the location of the rainfall transect.*

While a non-linear threshold-response of grass cover to rain seems to occur for the rain-gauge interpolated data, the response is more continuous for the originally coarser satellite-based observations (Figs. S4.1 and S4.2). Hence, the existence and identification of non-linearity and bistability in space-for-time approaches – important clues for regime shifts – may depend on the selected driver dataset. Moreover, the satellite-based rainfall estimates are higher, resulting in potentially late announcement of regime shifts as compared to the rain-gauge based dataset (Fig. S4.1). It seems reasonable to expect that the ground-based observations provide a more reliable rainfall estimate than the relatively coarse TRMM data. Nevertheless, the differential response between rainfall products may also originate from the interpolation procedure used or difference in time period covered. In addition, both in data-scarce regions and in larger scale analyses, availability of rainfall data may be restricted to satellite-based observations.

To better compare the type of response among datasets (Fig. S4.3), rain estimates are rescaled between zero and one (Eqn. S1), where R is estimated mean annual rainfall for a specific snapshot, and subscripts *min*, *max*, and *r* represent minimum, maximum, and rescaled rainfall.

$$R_r = \frac{R - R_{min}}{R_{max} - R_{min}} \qquad (Eqn. S1)$$

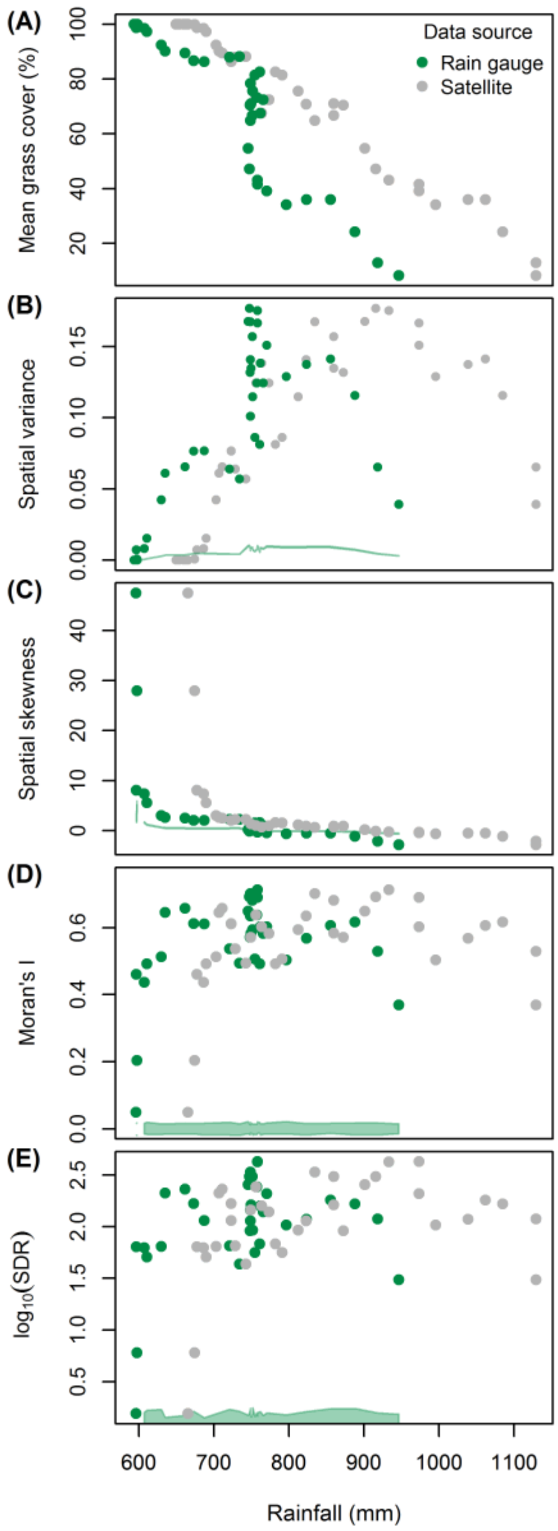

Figure S4.2. Rainfall dataset controls relationships between rainfall, grass cover, and onset of spatial early warning signals along spatial rainfall gradients in the Serengeti-Mara ecosystem. (A) *Mean grass cover response to rainfall* ***(B)*** *Spatial variance* ***(C)*** *Spatial skewness* ***(D)*** *Moran's I spatial correlation (value of 1 indicates perfect spatial correlation)* ***(E)*** *Logarithm (base 10) of the Spectral Density ratio (SDR). Spatial EWS were calculated conform (Kéfi et al., 2014), based on 40 snapshots (2.5 × 2.5 km) of forest-grass patterns (Reed, Anderson, Dempewolf, Metzger, & Serneels, 2008) along rainfall transect 5 in Eby, Agrawal, Majumder, Dobson, and Guttal (2017). Green shaded areas represent 95% confidence intervals of a null model without spatial structure (by randomly reshuffling (n = 99) the vegetation data). Rain-gauge interpolated mean annual rainfall sum data (1960 – 2004) were provided by Dr. M. Coughenour, satellite data (1998 – 2012) are downscaled from the Tropical Rainfall Measuring Mission dataset (Huffman et al., 2007). With reducing rainfall, spatial EWS start to rise (i.e. indicate an impending regime shift) at higher rain amounts for the satellite-based rainfall data.*

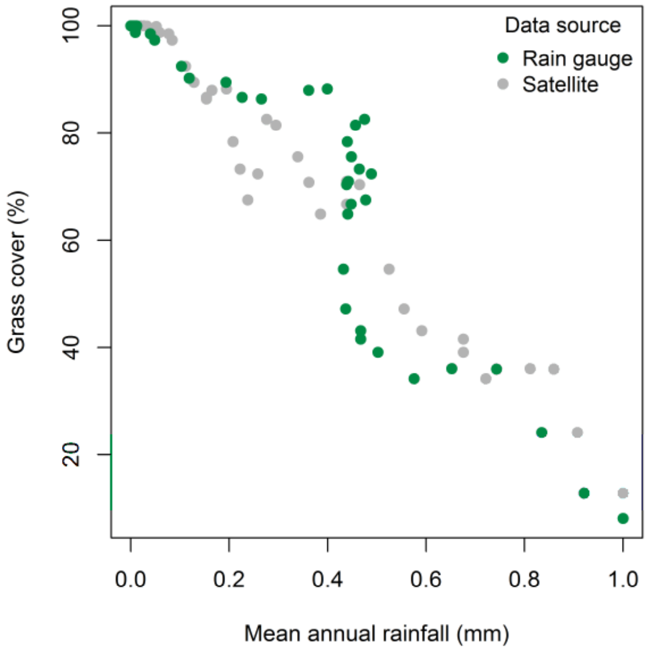

Figure S4.3. *Non-linear response of grass cover to rainfall based on on-site rain-gauge observations as compared to a more continuous response for rainfall based on satellite observations.*

S5: Variograms as spatial early warning signals

A (very) brief theory on variograms

In geostatistics, spatial heterogeneity is generally quantified using the variogram. Briefly, variogram analysis starts from the principle that the variability between points separated in space is small at small separation distances, and increases with larger distance between the points (Journel & Huijbregts, 1978). At some distance, points become uncorrelated in space and the variability (semivariance) stabilizes (Fig. 6 main text).

By calculating the mean semivariance for all point combinations and plotting it against the separation distance, one obtains the empirical variogram. Next, a variogram model can be fitted through the empirical variogram data to quantify spatial structure. Numerous variogram models are available, but have generally three parameters in common: the nugget, partial sill, and correlation range (Fig. 6 main text). The *correlation range* is a measure of the characteristic length scale at which spatial patterns emerge, or mean patch size (Woodcock, Strahler, & Jupp, 1988). The *nugget* is the random contribution to spatial variability, originating from spatial processes occurring at spatial scales smaller than the minimum separation distance or measurement error. The *partial sill* refers to the structured component of spatial variability. Together, the nugget and partial sill constitute the *sill*, the total spatial variability. Without spatial patterns, i.e. random noise, only a nugget effect occurs. In addition, one can assess whether patterns are anisotropic by selecting point pairs only in specific directions and fit variogram models to these subsets (Journel & Huijbregts, 1978).

Application

To test the value of variograms as spatial early warning signals, we subjected the snapshots created in the model simulations of the Noy-Meir model as presented in Box 1 and S1 to variogram analysis. The same snapshots were used to calculate regular spatial early warning signal analysis conform (Kéfi et al., 2014).

An important notion in variogram analysis is that the spatial process of interest is second order stationary. This implies that the mean and variance are space-invariant, and that the covariance between two points in space only depends on the separation distance between the two points (Journel & Huijbregts, 1978). In practice, often a quadratic or cubic trend surface is fitted through the spatial data to remove spatial trends in the mean and the residuals are used in variogram analysis (Oliver & Webster, 2014). Given the simulation data have no structured spatial component, no trend removal was applied.

Though a multitude of variogram models can be fitted, we fixed the variogram models to the ‘Spherical’ variant, thereby avoiding varying theoretical variogram model to affect the range parameter. We fitted the spherical variogram models using the INTAMAP package in R (Pebesma et al., 2011; R Core Team, 2018). No structured spatial variability was incorporated

in the Noy-Meir model, and diffusion was homogeneous and isotropic (S1). Testing for anisotropy (directionality of patterning) thus was deemed unnecessary.

Similarity between variogram parameters and contemporary spatial EWS

Although the *sill* is calculated differently as the spatial variance, its values are highly similar (Box 1; Fig. 6). Indeed, the sill is theoretically equal to the population variance (Barnes, 1991). The advantage of the variogram-based sill, however, is that it does not depend on the mean field value for categorical data, which is a known shortcoming of spatial variance (Sankaran, Majumder, Kéfi, & Guttal, 2017). The *relative structural variance* (100% · partial sill / (partial sill + nugget)) is comparable to Moran's I index for spatial autocorrelation (Moran, 1950), but gives quantitative insight in the proportion of spatially structured versus spatially random processes. The *correlation range*, an estimate of mean patch size (Woodcock et al., 1988), is comparable to wavelength analyses using e.g. the Discrete Fourier Transform (Carpenter & Brock, 2010). However, analyses of shifting wavelength spectra upon reduced ecosystem resilience have so far remained qualitatively (e.g. (Carpenter & Brock, 2010) and (Kéfi et al., 2014)). In contrast, the correlation range provides changes in patchiness in a single quantitative and easily interpretable metric that can be readily measured and validated in the field.

References in Supporting Information

- Barnes, R. J. (1991). The variogram sill and the sample variance. *Mathematical Geology, 23*(4), 673-678. doi: 10.1007/bf02065813
- Carpenter, S. R., & Brock, W. A. (2010). Early warnings of regime shifts in spatial dynamics using the discrete Fourier transform. *Ecosphere, 1*(5), 1-15. doi: 10.1890/es10-00016.1
- Coughenour, M. B. (1992). Spatial modeling and landscape characterization of an African pastoral ecosystem: a prototype model and its potential use for monitoring drought *Ecological Indicators* (pp. 787-810): Springer.
- Eby, S., Agrawal, A., Majumder, S., Dobson, A. P., & Guttal, V. (2017). Alternative stable states and spatial indicators of critical slowing down along a spatial gradient in a savanna ecosystem. *Global Ecology and Biogeography, 26*(6), 638-649. doi: 10.1111/geb.12570
- Guttal, V., & Jayaprakash, C. (2009). Spatial variance and spatial skewness: leading indicators of regime shifts in spatial ecological systems. *Theoretical Ecology, 2*(1), 3-12. doi: 10.1007/s12080-008-0033-1
- Hilbert, D. W., Roulet, N., & Moore, T. (2000). Modelling and analysis of peatlands as dynamical systems. *Journal of Ecology, 88*(2), 230-242. doi: 10.1046/j.1365-2745.2000.00438.x
- Hirota, M., Holmgren, M., Van Nes, E. H., & Scheffer, M. (2011). Global Resilience of Tropical Forest and Savanna to Critical Transitions. *Science, 334*(6053), 232.
- Huffman, G. J., Bolvin, D. T., Nelkin, E. J., Wolff, D. B., Adler, R. F., Gu, G., . . . Stocker, E. F. (2007). The TRMM Multisatellite Precipitation Analysis (TMPA): Quasi-Global, Multiyear, Combined-Sensor Precipitation Estimates at Fine Scales. *Journal of Hydrometeorology, 8*(1), 38-55. doi: 10.1175/jhm560.1
- Journel, A. G., & Huijbregts, C. J. (1978). *Mining Geostatistics*. Londen, UK: Academic press inc.
- Kéfi, S., Guttal, V., Brock, W. A., Carpenter, S. R., Ellison, A. M., Livina, V. N., . . . Dakos, V. (2014). Early warning signals of ecological transitions: methods for spatial patterns. *PLoS ONE, 9*(3), e92097.
- Moran, P. A. (1950). Notes on continuous stochastic phenomena. *Biometrika, 37*(1/2), 17-23.
- Noy-Meir, I. (1973). Desert Ecosystems: Environment and Producers. *Annual Review of Ecology and Systematics, 4*, 25-51.
- Oliver, M., & Webster, R. (2014). A tutorial guide to geostatistics: Computing and modelling variograms and kriging. *Catena, 113*, 56-69.
- Pebesma, E., Cornford, D., Dubois, G., Heuvelink, G. B., Hristopulos, D., Pilz, J., . . . Skøien, J. O. (2011). INTAMAP: the design and implementation of an interoperable automated interpolation web service. *Computers & Geosciences, 37*(3), 343-352.
- R Core Team. (2018). R: A language and environment for statistical computing. Vienna, Austria: R Foundation for Statistical Computing. Retrieved from http://www.R-project.org/
- Reed, D. N., Anderson, T. M., Dempewolf, J., Metzger, K., & Serneels, S. (2008). The spatial distribution of vegetation types in the Serengeti ecosystem: the influence of rainfall and topographic relief on vegetation patch characteristics. *Journal of Biogeography, 36*(4), 770-782. doi: doi:10.1111/j.1365-2699.2008.02017.x
- Rietkerk, M., Boerlijst, Maarten C., van Langevelde, F., HilleRisLambers, R., de Koppel, Johan v., Kumar, L., . . . de Roos, André M. (2002). Self-Organization of

Vegetation in Arid Ecosystems. . *The American Naturalist, 160*(4), 524-530. doi: 10.1086/342078
- Sankaran, S., Majumder, S., Kéfi, S., & Guttal, V. (2017). Implications of being discrete and spatial for detecting early warning signals of regime shifts. *Ecological Indicators*. doi: https://doi.org/10.1016/j.ecolind.2017.11.040
- Webster, R., & Oliver, M. A. (2007). *Geostatistics for Environmental Scientists*. Chichester, England: John Wiley & Sons.
- Woodcock, C. E., Strahler, A. H., & Jupp, D. L. B. (1988). The use of variograms in remote sensing: I. Scene models and simulated images. *Remote Sensing of Environment, 25*(3), 323-348. doi: https://doi.org/10.1016/0034-4257(88)90108-3
